# Levosimendan in Decompensated Heart Failure with Reduced Ejection Fraction in Older Adults: A Systematic Review of Safety and Efficacy

**DOI:** 10.3390/medicines12040023

**Published:** 2025-09-30

**Authors:** Esteban Zavaleta-Monestel, Jeaustin Mora-Jiménez, Kevin Cruz-Mora, Ernesto Martinez-Vargas, José Pablo Díaz-Madriz, Sebastián Arguedas-Chacón, Abigail Fallas-Mora, Carlos Wu-Chin, Jose Miguel Chaverrí-Fernandez

**Affiliations:** 1 Health Research Department, Clínica Bíblica, San José 1307-1000, Costa Rica; sarguedas@clinicabiblica.com; 2 Pharmacy Department, Clínica Bíblica, San José 1307-1000, Costa Rica; jmoraj@clinicabiblica.com (J.M.-J.); kevin.cruzmora@ucr.ac.cr (K.C.-M.); emartinezv@clinicabiblica.com (E.M.-V.); jdiazm@clinicabiblica.com (J.P.D.-M.); abigail.fallas11@gmail.com (A.F.-M.); 3 Pharmacy Department, University of Costa Rica, San José 11501-2060, Costa Rica; jose.chaverri@ucr.ac.cr; 4 Department of Critical Medicine and Intensive Care, Clínica Bíblica, San José 1307-1000, Costa Rica; carloswuchin@yahoo.com

**Keywords:** levosimendan, decompensated heart failure, older adults, geriatrics, safety, efficacy, heart failure with reduced ejection fraction

## Abstract

Background/Objectives: Heart failure with reduced ejection fraction (HFrEF) is a leading cause of hospitalization and functional decline in older adults, accounting for over 80% of all heart failure cases. Given the narrow therapeutic window of currently available inotropes and the vulnerability of this population, levosimendan has been proposed as a potential alternative. This systematic review aimed to evaluate the clinical efficacy and safety of levosimendan in older adults with decompensated HFrEF. Methods: A systematic search of PubMed, Embase, Scopus, and the Cochrane Library was conducted between January and May 2025, following PRISMA 2020 guidelines. The review was registered in PROSPERO (CRD420251032329). Of 379 articles initially identified, 8 studies (randomized, observational, and single-arm designs) enrolling patients aged ≥65 years with decompensated HFrEF met the inclusion criteria. Study quality was assessed using the Cochrane RoB-2 tool and JBI Critical Appraisal Checklists. No meta-analysis was performed due to heterogeneity in study designs, populations, and interventions. Results: A total of 2838 patients were analyzed. Levosimendan was associated with short-term improvements in hemodynamic parameters, including an increase in cardiac index (from 1.65 to 2.37 L/min/m^2^) and a reduction in pulmonary capillary wedge pressure (from 31 to 16 mmHg) within 24–72 h (*p* < 0.002). However, no statistically significant differences were observed in 30-, 90-, or 180-day mortality (*p* > 0.05), and findings on rehospitalization were inconsistent. Reported adverse events included hypotension (36–57%) and atrial arrhythmias (9–50%), with low treatment discontinuation rates (5–8%). Conclusions: Levosimendan may improve short-term hemodynamic parameters in older adults with decompensated HFrEF, but the available evidence is limited and heterogeneous. Its effects on mortality and rehospitalization remain inconclusive. Clinical use should be individualized and closely monitored, particularly in frail patients.

## 1. Introduction

Heart failure (HF) is a clinical syndrome characterized by the heart’s inability to pump blood efficiently to meet the body’s metabolic demands. It is classified according to left ventricular ejection fraction (LVEF) into reduced, preserved, or mildly reduced categories, and by clinical presentation as acute or chronic [1]. In heart failure with reduced ejection fraction (HFrEF), LVEF is <40%, and patients often experience symptoms refractory to optimal medical therapy, recurrent episodes of decompensation, functional decline, and high hospitalization rates [2].

HFrEF predominantly affects older adults, with over 80% of cases occurring in individuals aged ≥65 years and an incidence close to 10 per 1000 persons per year. Its progression is often accompanied by hemodynamic deterioration and neurohormonal activation, leading to common comorbidities such as renal insufficiency, chronic obstructive pulmonary disease (COPD), and anemia. Moreover, currently available inotropic agents have a narrow therapeutic window and carry a substantial risk of arrhythmias, further complicating pharmacological management in this population [2].

The use of levosimendan in older adults requires a carefully individualized clinical approach due to their increased susceptibility to hemodynamic and arrhythmic adverse events. In patients with acute or decompensated HF, levosimendan has been shown to improve clinical stability and hemodynamic parameters without significantly increasing the incidence of serious complications. Nevertheless, administration should always be accompanied by close monitoring to ensure safety [3,4].

Although current evidence offers a general understanding of HFrEF management, it remains insufficient to guide therapy specifically in older adults. Therefore, this systematic review aims to evaluate the impact of levosimendan in patients aged ≥65 years with decompensated HFrEF, comparing it with standard care—either without inotropes or with alternative inotropic agents—and analyzing outcomes related to efficacy (hemodynamic function, mortality, rehospitalization) and safety (symptomatic hypotension, arrhythmias).

## 2. Methods

This systematic review was conducted following the recommendations of the Preferred Reporting Items for Systematic Reviews and Meta-Analyses (PRISMA) 2020 guidelines [5]. Additional details, including the PRISMA 2020 Checklist and the full search strategies for each database, are provided in the Supplementary Materials.

### 2.1. Protocol Registration

The protocol was registered in the PROSPERO database (International Prospective Register of Systematic Reviews) under registration number CRD420251032329. The full registration record is publicly available at: https://www.crd.york.ac.uk/PROSPERO/view/CRD420251032329 (accessed on 16 September 2025).

### 2.2. Search Strategy

A comprehensive search was performed in PubMed, MEDLINE, Embase, Scopus, and the Cochrane Library to identify relevant studies evaluating levosimendan in older adults with decompensated heart failure with reduced ejection fraction (HFrEF).

The search strategy combined controlled vocabulary (MeSH terms) and free-text terms with Boolean operators (AND, OR) to maximize sensitivity. The keywords included: “Levosimendan”, “Simdax”, “Heart Failure, Systolic”, “Heart Failure with Reduced Ejection Fraction”, “HFrEF”, “Older Adults”, “Elderly”, “Geriatric Patients”, “Patients over 65 years”, “Clinical Trial”, “Randomized Controlled Trial”, “Observational Study”, “Hemodynamics”, “Cardiac Output”, and “Left Ventricular Function”. Searches were restricted to articles published between January 2005 and May 2025. The detailed search strategies for each database are provided in Appendix A.

### 2.3. Eligibility Criteria

This systematic review was conducted in accordance with the PRISMA statement and structured around the PICO question. Older adults with heart failure with reduced ejection fraction (HFrEF) were included as the population (P), assessing the administration of levosimendan (I) in comparison with other pharmacological treatments such as dobutamine, milrinone, or no inotropes (C). The outcomes analyzed (O) were improvement in cardiac function, reduction in mortality, hospitalizations, and adverse events. The research question posed was: In older adults with HFrEF, does levosimendan improve clinical outcomes compared to standard treatment?

#### 2.3.1. Inclusion Criteria

Articles written entirely in English and published within the last 20 years were selected, including patients with decompensated heart failure with reduced ejection fraction (HFrEF). Studies with participants over 18 years of age were accepted, provided the population was representative of a geriatric profile, defined as having a mean age of 65 years or older. The search was limited to clinical trials, analytical observational studies, systematic reviews, and meta-analyses.

#### 2.3.2. Exclusion Criteria

Studies that did not specify the age of participants were excluded. Additionally, studies involving patients with heart failure with preserved ejection fraction (HFpEF), as well as those with incomplete or inaccessible data, were also excluded. Any discrepancies between reviewers were resolved by a third investigator through analysis and discussion.

### 2.4. Study Selection and Documentation

Two reviewers independently screened titles and abstracts for eligibility. Full texts of potentially relevant studies were retrieved and assessed against the inclusion criteria. Reference lists of selected articles and relevant reviews were manually screened to identify additional eligible studies. Any disagreements were resolved through discussion with a third reviewer

### 2.5. Risk of Bias

Data collection was carried out using a predesigned table developed before evaluating the selected articles. Two independent reviewers assessed the methodological quality of the eligible studies and reached a consensus. For randomized clinical trials, the Cochrane Risk of Bias Tool (RoB-2) was used, which evaluates seven domains related to study design, conduct, and reporting. For non-randomized studies (observational and single-arm), the Joanna Briggs Institute (JBI) Critical Appraisal Tool was applied, using the appropriate version to each study design. Any discrepancies between reviewers were resolved by consensus or with the intervention of a third evaluator.

## 3. Results

### 3.1. Study Selection

**Figure 1:**
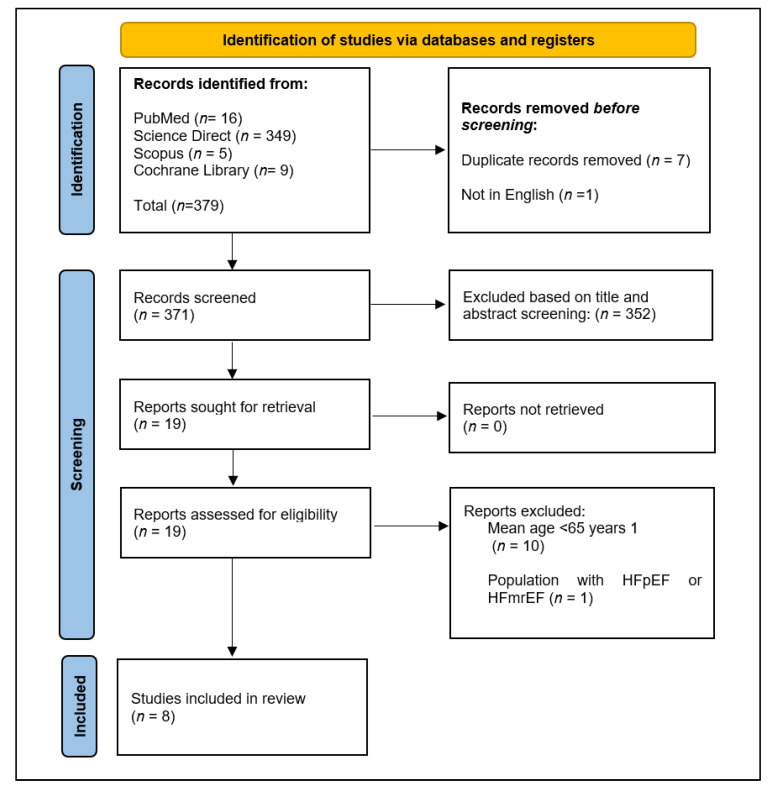
Flow diagram of the study selection process according to the PRISMA 2020 statement.

Figure 1 illustrates the study selection process. A total of 379 records were initially identified through comprehensive searches in PubMed (n = 16), ScienceDirect (n = 349), Scopus (n = 5), and the Cochrane Library (n = 9). Before screening, 7 duplicate records were removed, along with 1 article written in a language other than English, resulting in 371 unique records for title and abstract screening. During this phase, 352 records were excluded because they did not meet the predefined eligibility criteria, which included population characteristics, study design, and relevance to the research question. Nineteen full-text articles were subsequently retrieved and assessed for eligibility. Of these, 10 were excluded because the mean age of participants was below 65 years, and 1 was excluded because the study population comprised patients with heart failure with preserved ejection fraction (HFpEF) or heart failure with mildly reduced ejection fraction (HFmrEF), conditions outside the scope of the review. Consequently, 8 studies fulfilled all inclusion criteria and were incorporated into the final systematic review.

### 3.2. Characteristics of the Included Studies

Table 1 summarizes the clinical characteristics and main outcomes of the included studies. Eight studies were included, comprising predominantly randomized controlled trials, along with one observational study and one single-arm interventional study. The populations evaluated varied, including older adults with decompensated HFrEF as well as patients undergoing cardiac surgery with reduced LVEF. Interventions assessed levosimendan either in comparison with standard care, placebo, or alternative inotropes such as dobutamine. Outcomes spanned hemodynamic parameters (cardiac index, pulmonary capillary wedge pressure), clinical endpoints (all-cause mortality, rehospitalization), and safety events (hypotension, arrhythmias). Given the heterogeneity in study designs, populations, and outcome measures, no meta-analysis was performed, and results are presented descriptively without pooled quantitative synthesis.

### 3.3. Primary Outcomes

#### 3.3.1. Mortality and Hospitalization

As shown in Table 2, studies on HFrEF do not demonstrate clear mortality benefits from levosimendan. Mebazaa et al. (2007) [7], Cholley et al., 2017 [10] and Mehta et al., 2017 [11] found no significant differences in survival at 30, 90, or 180 days, nor in comparisons at 28 and 180 days (*p* values between 0.12 and 0.59). In García-González et al. (2015) [9] reported a 75% 30-day survival rate following aortic valve replacement; however, the lack of a control group and insufficient statistical power preclude attributing this outcome to the drug.

Following discharge, Pölzl et al. (2023) [12] also found no reduction in decompensation events through week 14 (*p* = 0.064), and even recorded a higher number of cardiovascular events in the levosimendan arm. Lastly, Visco et al. (2024) [13] reported a 50% reduction in hospitalizations and a 68% decrease in inpatient days using CardioMEMS and on-demand levosimendan administration; however, the absence of statistical validation and the small sample size limit the reliability of these findings.

#### 3.3.2. Cardiac Function and Hemodynamics

As shown in Table 2, Levosimendan may exert an acute and sustained effect on hemodynamic parameters in older adults with HFrEF. For instance, Adamopoulos et al. 2006 [6] reported that, following a single 72 h infusion of levosimendan, pulmonary capillary wedge pressure (PCWP) decreased from 24 ± 3 to 19 ± 2 mmHg, cardiac index (CI) increased from 1.7 ± 0.4 to 1.9 ± 0.5 L/min/m^2^, and left ventricular ejection fraction (LVEF) improved from 24 ± 5% to 28 ± 6% (*p* < 0.05 for all parameters).

Similarly, Bergh et al. (2010) [8] observed at 48 h a reduction in PCWP of 8.3 ± 6.7 mmHg and an increase in CI of 0.66 ± 0.63 L/min/m^2^; they also reported a mean decrease in NT-proBNP of 507 ng/mL (*p* ≤ 0.05). Likewise, In et al. (2015) [9] found that at 24 h, CI increased from 1.65 ± 0.20 to 2.37 ± 0.49 L/min/m^2^, PCWP dropped from 31 ± 10 to 16 ± 4 mmHg, and stroke volume index (SVI) rose from 21.1 ± 7.1 to 30.1 ± 7.4 mL/m^2^ (*p* ≤ 0.002 in all cases), findings are consistent with a vasodilatory and inotropic profile.

### 3.4. Secondary Outcomes

#### 3.4.1. Adverse Events (AE)

As shown in Table 3, hypotension was the most frequently reported adverse effect with levosimendan, affecting 35–57% of patients compared to 7–48% in the comparator groups [8,9,10,12]. Arrhythmias, particularly atrial fibrillation (AF), were observed in a wide range (9–50% vs. 6–40%), although several studies did not show significant differences [8,11,12]. Other events such as hypokalemia (6–9%), headache (5–8%), third-degree atrioventricular block (4% vs. 9%), or stroke (2.4–3.5%) were infrequent, indicating an overall acceptable tolerability profile, except for the need to monitor blood pressure and heart rhythm closely [7,10,11].

#### 3.4.2. Treatment Discontinuations

As shown in Table 3, levosimendan discontinuation due to adverse events was infrequent, occurring in 5% to 8% of treated patients compared to 3–4% in control groups (Cholley et al., 2017 [10] Mehta et al., 2017 [11], with no statistically significant differences in patients undergoing cardiopulmonary bypass surgery. The fact that other studies did not report discontinuation rates further supports the overall acceptable tolerability of the drug, despite the incidence of hypotension and arrhythmias.

### 3.5. Methodological Quality

#### 3.5.1. Assessment Using the ROB-2 Tool

**Figure 2:**
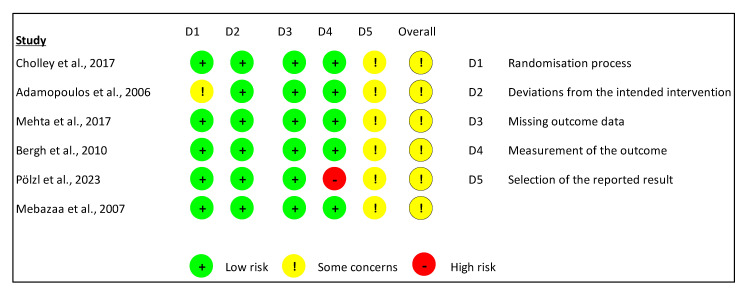
Risk of Bias Assessment of the Six Included Studies Using the ROB-2 Tool (Domains D1–D5 and Overall Judgment). References: Adamopoulos et al., 2006 [6]; Mebazaa et al., 2007 [7]; Bergh et al., 2010 [8]; Mehta et al., 2017 [11]; Cholley et al., 2017 [10]; Pölzl et al., 2023 [12].

Two reviewers (J.M-J and K.C-M) independently assessed the risk of bias and study quality. Figure 2 presents the evaluation of six randomized clinical trials using the RoB-2 tool. All studies appropriately managed deviations from the intended interventions and missing outcome data (Domains D2–D3). However, Adamopoulos et al. [6] showed weaknesses in the randomization process (D1), and Pölzl et al. [12] raised concerns regarding the reliability of outcome measurements (D4). Additionally, all studies presented inconsistencies in the selection of reported outcomes (D5). Consequently, each trial received a global judgment of moderate risk of bias, which may reduce the overall confidence in the findings.

#### 3.5.2. Evaluation Using the JBI Critical Appraisal Tool

**Figure 3:**
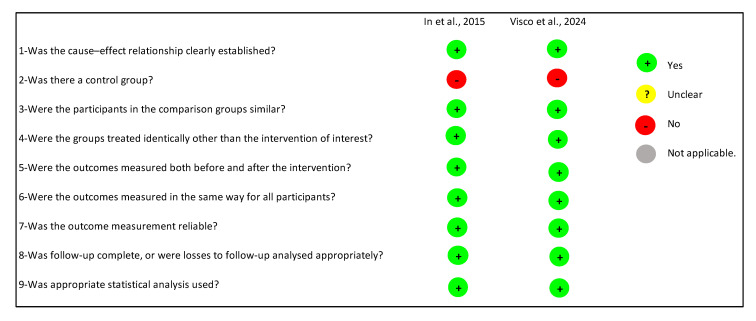
Methodological Quality Assessment of Prevalence Studies Using the JBI Critical Appraisal Tool for Studies Reporting Prevalence Data. References: In et al., 2015 [9]; Visco et al., 2024 [13].

In Section 3.5.2, two reviewers (J.M.-J. and K.C.-M.) applied for the JBI Critical Appraisal Tool for Quasi-Experimental Studies, which includes nine domains to assess methodological quality. Based on these criteria, no study received a score indicative of low quality (0–4 domains), while both articles were rated as high quality, fulfilling between 5 and 9 domains. Details of these assessments are presented in Figure 3.

### 3.6. Summary of Findings for the Prespecified Outcomes and Certainty of the Evidence (GRADE)

As shown in Table 4, the evidence synthesis indicates that levosimendan, compared with standard care without inotropes or with alternative inotropes, probably results in a small to no difference in mortality (low certainty) and may be associated with a slight increase in hypotension (moderate certainty). The effect on arrhythmias is uncertain, with a possible small to no difference (low certainty). Levosimendan probably reduces pulmonary capillary wedge pressure (moderate certainty) but shows little to no effect on cardiac index at 48 h (moderate certainty). For hospitalization outcomes, the evidence is very uncertain (very low certainty) due to reliance on a single observational study at high risk of bias and potential confounding. Hemodynamic outcomes from a single observational study with nine participants were not included in the SoF table due to very low certainty and limited applicability; these results are described narratively in the main text.

## 4. Discussion

### 4.1. Effectiveness of Levosimendan in Decompensated Heart Failure in Older Adults

#### 4.1.1. Mortality and Hospitalizations

Several studies in geriatric and surgical populations have assessed the impact of levosimendan on mortality, yielding inconsistent results. In older adults with HFrEF, Mebazaa et al. (2007) and Pölzl et al. (2023), after propensity score adjustment in critically ill patients, reported no significant differences at 30, 90, or 180 days (*p* > 0.05) [7,12,14]. In patients undergoing cardiopulmonary bypass surgery, Cholley et al. (2017) and Mehta et al. (2017) also found no mortality benefit. Conversely, a meta-analysis by Landoni et al. (2018) demonstrated a significant reduction in perioperative mortality following CABG (RR 0.45; 95% CI: 0.29–0.71; *p* = 0.0005), but no effect in patients undergoing valvular surgery (RR 0.64; 95% CI: 0.12–3.38; *p* = 0.60) [10,11,15]. These discrepancies may be explained by differences in study design, patient heterogeneity, and variations in drug administration protocols

Although evidence in older adults with decompensated heart failure does not consistently show a benefit in reducing rehospitalizations [12]. The literature is generally heterogeneous. A meta-analysis of 45 randomized trials (n = 5480) reported that levosimendan reduced hospital length of stay by –1.31 days (95% CI: –1.95 to –0.31; *p* = 0.007), although substantial heterogeneity was observed (I^2^ = 71%) [16]. Overall, these findings suggest that while levosimendan may shorten hospital stays, its effects on mortality and rehospitalizations remain inconsistent, and its use should be individualized based on each patient’s clinical profile.

#### 4.1.2. Hemodynamic Effects

In older adults, levosimendan exhibits both vasodilatory and inotropic properties, leading to measurable improvements in PCWP, CI, LVEF, and NT-proBNP levels, sustained for periods ranging from 24 to 72 h [6,8,9]. These results are consistent with evidence from several meta-analyses showing that both inpatient and outpatient infusions are associated with an approximate increase in cardiac output of 0.6–0.7 L/min/m^2^, along with improvements in multiple hemodynamic markers. Notably, in post-surgical patients, these hemodynamic benefits may persist for up to 7 days [17,18].

**Figure 4:**
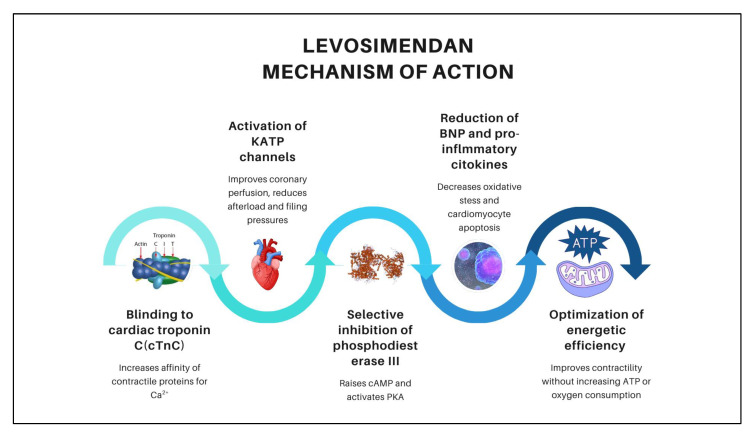
Mechanisms of Action of Levosimendan and Its Hemodynamic Effects.

The hemodynamic benefits of levosimendan in improving cardiac performance and myocardial perfusion arise from five synergistic mechanisms of action, as illustrated in Figure 4. First, it binds to cardiac troponin C (cTnC), increasing the affinity of contractile proteins for Ca^2+^, thereby enhancing inotropy without elevating myocardial oxygen consumption or intracellular calcium levels [19]. Second, it activates adenosine triphosphate (ATP)-sensitive potassium (KATP) channels in the sarcolemma of vascular smooth muscle, improving coronary perfusion, reducing afterload, and lowering filling pressures. Simultaneously, activation of mitochondrial KATP channels exerts cardioprotective, anti-ischemic, anti-inflammatory, and anti-apoptotic effects [20]. Third, selective inhibition of phosphodiesterase III in the myocardium increases cyclic adenosine monophosphate (cAMP) levels, activates protein kinase A (PKA), and induces phosphorylation of L-type calcium channels and phospholamban, enhancing both calcium influx during action potentials and its reuptake into the sarcoplasmic reticulum—producing potent inotropic and lusitropic effects [21].

Additionally, levosimendan reduces circulating levels of brain natriuretic peptide (BNP) and pro-inflammatory cytokines, attenuates oxidative stress, and prevents cardiomyocyte apoptosis. By optimizing contractility without increasing ATP consumption or oxygen demand, it enhances energetic efficiency and consolidates its therapeutic value in the management of decompensated heart failure in older adults [22].

### 4.2. Security of Levosimendan in Decompensated Heart Failure in Older Adults

#### 4.2.1. Tolerability and Adverse Effects of Levosimendan in Decompensated Heart Failure in Older Adults

When administering levosimendan to older adults, potential adverse reactions must be carefully anticipated and monitored. Across the reviewed studies, hypotension emerged as the most frequent adverse event, reported in 36–57% of patients [8,10,11]. In the REVIVE II trial, which included patients aged ≥65 years hospitalized for acute decompensated heart failure, hypotension occurred in 50.2% of participants and was the leading cause of treatment discontinuation [23].

The second most common adverse event was arrhythmia, particularly atrial fibrillation and ventricular tachycardia, with incidence rates ranging from 9% to 50% [8,12,15]. Some studies reported atrial fibrillation in up to 14% of patients, noting that most episodes were brief and clinically manageable [24]. Additional adverse events—including headache, nausea, hypokalemia, and stroke—were infrequent. Overall, while the incidence of serious adverse events in this population is relatively low, close hemodynamic and electrocardiographic monitoring is warranted throughout treatment [25].

#### 4.2.2. Considerations and Precautions for the Use of Levosimendan in Decompensated Heart Failure in Older Adults

In advanced heart failure with reduced ejection fraction (advHFrEF), levosimendan may be considered as a palliative intervention or as a strategy for transient hemodynamic improvement in cases with clinical or hemodynamic evidence of organ hypoperfusion. The initial infusion should be administered in a hospital setting, starting at 0.2 µg/kg/min over 24 h, to ensure safety and detect potential complications such as symptomatic hypotension or ventricular tachycardia. For palliative use, clinical benefit is primarily assessed through subjective improvement in symptoms and quality of life, which can be complemented by reductions in NT-proBNP levels. In cases aimed at optimizing hemodynamics before advanced therapies (e.g., LVAD implantation), assessment may include echocardiography or right heart catheterization [19].

In patients with systolic blood pressure > 100 mmHg and mildly reduced renal function (estimated glomerular filtration rate [eGFR] > 45 mL/min/1.73 m^2^), intermittent dosing of 6.25 mg every two weeks is suggested. In those with systolic blood pressure < 100 mmHg, significantly impaired renal function (eGFR < 45 mL/min/1.73 m^2^), or a history of complex ventricular arrhythmias, the regimen should be adjusted to 12.5 mg every four weeks [26].

In patients scheduled for LVAD implantation, levosimendan should be administered 24 h before surgery at 0.2 µg/kg/min, with the addition of norepinephrine or epinephrine (0.1–0.2 µg/kg/min) if the risk of post-implant right ventricular dysfunction is high. Dosing should be individualized according to the patient’s clinical profile and treatment response, with close monitoring due to the increased risk of adverse effects in older adults with multiple comorbidities [27].

#### 4.2.3. Practical Precautions and Titration Guidelines for Levosimendan in Geriatric Decompensated Heart Failure

Levosimendan can be administered at doses ranging from 0.05 to 0.2 µg/kg/min over 24 h. Although traditionally preceded by a 6–12 µg/kg bolus, the use of a loading dose is no longer routinely recommended in clinical practice due to safety concerns. The most commonly employed regimen is a continuous infusion of 0.1 µg/kg/min without a bolus, as loading doses are associated with a higher risk of hypotension and arrhythmias, particularly in frail, hypovolemic, or hypotensive patients [28].

Current best practice favors starting the infusion at the target rate and adjusting gradually based on the patient’s hemodynamic response. The total infusion volume typically ranges from 3 to 12 mL, depending on dose and body weight. A slow, individualized titration is advised to minimize the risk of hypotension [28].

For preparation, one vial containing 12.5 mg (5 mL) of levosimendan should be diluted in 250 mL of 5% dextrose solution. Alternatively, two vials (25 mg total) can be diluted in 500 mL of the same diluent. Using 5% dextrose is essential to maintain solubility and chemical stability, preventing precipitation and infusion-related reactions. Other diluents, such as saline or Ringer’s lactate, are not recommended due to an increased risk of drug instability or precipitation [10,28].

### 4.3. Strengths and Limitations

This systematic review has several strengths, including a comprehensive and reproducible search strategy across multiple databases, adherence to PRISMA 2020 guidelines, and the use of validated tools (RoB-2 and JBI) for quality assessment. The inclusion criteria ensured a focus on older adults (mean age ≥65 years), addressing a clinically relevant but often underrepresented population in heart failure research.

However, important limitations must be acknowledged. The evidence base is limited by the small number of studies specifically designed for older adults with HFrEF, and the substantial heterogeneity in study design, patient populations, intervention protocols, and outcome measures precluded meta-analysis. Most trials were not powered to detect differences in hard clinical outcomes such as mortality or rehospitalization, and several presented moderate risk of bias, particularly regarding allocation concealment, blinding, and selective reporting. The certainty of the evidence for several key outcomes was downgraded due to imprecision and indirectness.

These limitations restrict the generalizability of the findings and make the interpretation of results inherently cautious. Larger, multicenter, randomized controlled trials specifically designed for the geriatric HFrEF population are needed to clarify the potential role of levosimendan, particularly for clinical endpoints beyond transient hemodynamic improvement.

## 5. Conclusions

The use of levosimendan has not shown a consistent effect on survival in older adults with decompensated HFrEF at 30, 90, or 180 days. Additionally, no clear reduction was observed in rehospitalization rates or hospital length of stay in this population. However, the results suggest potential hemodynamic benefits from 24 to 72 h after administration, particularly in patients undergoing surgical intervention.

On the other hand, the most frequently reported adverse event was hypotension, occurring in up to 57% of cases. Transient arrhythmias such as atrial fibrillation and supraventricular tachycardia (SVT) were also observed; although manageable, these require close monitoring. Other adverse events were infrequent, but their occurrence in a clinically fragile population supports the need for rigorous monitoring.

Overall, this review provides relevant evidence for geriatric clinical practice by showing that although levosimendan does not appear to improve survival or reduce hospitalizations in older adults with HFrEF, it might have a role in selected clinical scenarios, such as palliative care as a potential action for transient hemodynamic improvement. However, this potential use should be interpreted with caution given the lack of consistent benefit in clinical outcomes. Its administration should occur in a hospital setting, with appropriate monitoring and individualized dose adjustments based on blood pressure and renal function.

## Figures and Tables

**Table 1:** Clinical Characteristics and Main Findings of the Included Studies.

Study	Study Type	Study Population	Average Age	Study Focus	Summary
Adamopoulos et al., 2006 [6].	Randomized controlled trial	69 patients with chronic decompensated heart failure, NYHA class III or IV, left ventricular ejection fraction (LVEF) ≤30%	Mean age: 67 years in the dobutamine group, 71 years in the levosimendan group, and 71 years in the placebo group.	Cardiac function parameters, inflammatory markers, event-free survival	Levosimendan improved hemodynamic parameters and reduced inflammatory and pro-apoptotic markers. It showed greater event-free survival compared to dobutamine and placebo
Mebazaa et al., 2007 [7].	Randomized, double-blind, multicenter trial	1327 patients with acute decompensated heart failure	Mean age: 67 years in the levosimendan group, 66 years in the dobutamine group	All-cause mortality at 180 days	The SURVIVE trial found no difference in survival between levosimendan and dobutamine, although levosimendan more effectively reduced BNP level
Bergh et al., 2010 [8].	Phase IV, randomized, double-blind study	60 patients with acute decompensated heart failure, NYHA class III-IV, LVEF < 35%	Mean age: 70 years in the levosimendan group, 71 years in the dobutamine group	Changes in cardiac index and pulmonary capillary wedge pressure	Levosimendan showed hemodynamic and neurohormonal improvement comparable to dobutamine at 24 h and superior at 48 h in decompensated HF patients receiving beta-blockers.
In et al., 2015 [9]	Single-arm interventional study	9 patients with severe aortic stenosis and reduced LVEF (≤40%)	Mean age 76 for levosimendan	Change in cardiac index	Levosimendan improved hemodynamic parameters in patients with severe aortic stenosis and reduced LVEF, serving as an effective bridge to valve replacement or vasodilator therapy
Cholley et al., 2017 [10].	Randomized, double-blind, placebo-controlled trial	336 patients with LVEF ≤ 40% undergoing coronary artery bypass grafting (CABG) with cardiopulmonary bypass	Mean age 69 for group of levosimendan vs. 67 placebo	Need for prolonged catecholamines, left ventricular assist device, or renal replacement therapy	Preoperative levosimendan did not significantly reduce postoperative low cardiac output syndrome in patients with low LVEF undergoing CABG.
Mehta et al., 2017 [11].	Randomized, placebo-controlled trial	849 patients with LVEF ≤ 35% undergoing cardiac surgery with cardiopulmonary bypass	Mean age 65 for group of levosimendan and placebo	Composite outcome of death, renal replacement therapy, perioperative myocardial infarction, or use of mechanical circulatory support	Prophylactic levosimendan did not reduce the rate of the composite outcome of death, renal replacement therapy (RRT), perioperative infarction, or mechanical support use in patients with reduced LVEF undergoing cardiac surgery with cardiopulmonary bypass.
Pölzl et al., 2023 [12].	Randomized, double-blind, placebo-controlled trial	148 patients with advanced chronic heart failure	Mean age 69.3 for group of levosimendan vs. 67.8 placebo	Global assessment including death, urgent heart transplant/VAD use, and change in NT-proBNP	The LeoDOR trial did not demonstrate improvement in clinical stability with intermittent levosimendan post-discharge in advanced HF due to increased cardiovascular events and low statistical power.
Visco et al., 2024 [13].	Observational study	7 patients with advanced heart failure who received a CardioMEMS implant (a pulmonary artery pressure monitoring system)	Mean age 69 for levosimendan	Hospitalizations, quality of life, echocardiographic parameters	Real-world study showed that CardioMEMS reduced hospitalizations and improved quality of life in HF patients by optimizing levosimendan use and generating economic benefits.

**Table 2:** Effects of Levosimendan on Hemodynamic Parameters, Mortality, and Hospitalization: Clinical Evidence.

Study	Cardiac Function/Hemodynamics	Mortality/Hospitalizations	Statistical Significance
Adamopoulos et al., 2006 [6].	Decrease in PCWP, improvement in CI and LVEF	Greater event-free survival with levosimendan	*p* < 0.05 for all hemodynamic parameters
Mebazaa et al., 2007 [7].	N/R	No significant difference in 180-day mortality vs. dobutamine	*p* = 0.40
Bergh et al., 2010 [8].	Decrease in PCWP, improvement in CI at 48 h	N/R	CI *p* = 0.037, PCWP *p* = 0.015, BNP *p* = 0.03
In et al., 2015 [9].	Improvement in CI and decrease in PCWP at 24 h. Decrease in SVI	75% 30-day survival observed	*p* < 0.05 for all hemodynamic parameters
Cholley et al., 2017 [10].	N/R	No significant difference in 180-day mortality	*p* = 0.15
Mehta et al., 2017 [11].	N/R	No significant difference in 90-day mortality	*p* = 0.98
Pölzl et al., 2023 [12].	N/R	No significant difference in mortality at 14 weeks	*p* = 0.064
Visco et al., 2024 [13].	N/R	68.7% reduction in hospitalization days and 50% reduction in total hospitalizations	N/R

Abbreviations: CI, cardiac index; LVEF, left ventricular ejection fraction; PCWP, pulmonary capillary wedge pressure; SVI, stroke volume index; BNP, B-type natriuretic peptide; N/R, not reported. Note: *p* < 0.05 considered statistically significant.

**Table 3:** Clinical Studies with Effectively Reported Secondary Outcomes.

Study	Hypotension	Arrhythmias	Other Adverse Events	Drug Discontinuation	Statistical Significance
Mebazaa et al., 2007 [7].	No significant difference	AF: 9.1% vs. 6.1% (dobutamine)	Hypokalemia: 9.4% vs. 5.9% (dobutamine), Headache: 8.3% vs. 4.7% (dobutamine)	N/R	*p* < 0.05 for all AEs
Bergh et al., 2010 [8].	35% vs. 7% (dobutamine)	No increase in AF or ventricular tachycardia (VT)	Nausea is more frequent with levosimendan	N/R	*p* < 0.05 for all AEs
Cholley et al., 2017 [10].	57% vs. 48% (placebo)	AF: 50% vs. 40% (placebo)	Third-degree AV block: 4% vs. 9% (placebo)	8% vs. 3% (placebo)	There is no significant difference in any AE
Mehta et al., 2017 [11].	36.2% vs. 32.8% (placebo)	AF: 38.1% vs. 33.0% (placebo), VT/VF: 10.7% vs. 9.7% (placebo)	Stroke: 3.5% vs. 2.4% (placebo)	Temporary interruption: 5.8% vs. 3.8% (placebo)	There is no significant difference in any AE
Pölzl et al., 2023 [12].	9.7% vs. 11.1% (placebo)	Trend toward more frequent arrhythmias (2.7% vs. 0.8% placebo)	N/R	N/R	There is no significant difference in any AE

Abbreviations: AF, atrial fibrillation; VT, ventricular tachycardia; VF, ventricular fibrillation; AV, atrioventricular; N/R, not reported; AE(s), adverse event(s). Note: *p* < 0.05 considered statistically significant.

**Table 4:** Summary of Findings (SoF) for the Prespecified Outcomes and Certainty of the Evidence (GRADE).

Outcome and Follow-Up	Nº Participants (Nº Studies and Type)	Effect (95% CI)	Absolute Effects			Certainty of Evidence (Quality of Evidence)	Plain Language Summary
			Standard Care Without Inotropes or with Alternative Inotropes (Dobutamine, Milrinone	**Levosimendan**	**Difference**		
Mortality	2656 (4 RCTs)	RR = 0.92 (0.73 to 1.18)	95 per 1000	87 per 1000	8 fewer per 1000 (26 fewer to 17 more)	⊕⊕◯◯ Low ^ab^	Low-certainty evidence suggests levosimendan probably results in little or no difference in mortality compared to standard care without inotropes or with alternative inotropes.
Hospitalizations	18 (1 observational)	In a small observational study, CardioMEMS monitoring combined with repeated levosimendan infusions was linked to fewer heart failure hospitalizations and hospitalization days, though the effect cannot be attributed solely to levosimendan due to co-intervention and the non-randomized design.				⊕◯◯◯ Very Low ^ac^	Very uncertain about the effect of levosimendan on hospitalizations; only one study at high risk of bias and possible confounding from CardioMEMS device.
Hemodynamics—Cardiac Index (48 h)	106 (2 RCTs)	----------	2.02 L/min/m^2^	2.20 L/min/m^2^	0.15 L/min/m^2^	⊕⊕⊕◯ Moderate ^a^	Levosimendan is likely to result in a small to no difference in hemodynamics (CI).
Hemodynamics (PCWP)	106 (2 RCTs)	-----------	20.9 mmHg	16.64 mmHg	−4.26 (−6.46 to −2.06)	⊕⊕⊕◯ Moderate ^a^	Levosimendan probably reduces pulmonary capillary wedge pressure (PCWP) compared to standard care.
Hypotension	1361 (4 RCTs)	RR = 1.13 (0.96 to 1.33)	401 per 1000	453 per 1000 (385 to 533)	52 more per 1000 (from 16 fewer to 132 more)	⊕⊕⊕◯ Moderate ^ab^	Levosimendan is likely to result in a slight increase in hypotension.
Arrhythmias	1359 (4 RCTs)	RR = 1.14 (0.98 to 1.32)	314 per 1000	358 per 1000 (308 to 415)	44 more per 1000 (from 6 fewer to 100 more)	⊕⊕◯◯ Low ^ab^	Levosimendan may result in a small to no difference in arrhythmias.

^a^ Downgraded one level due to risk of bias: some trials had unclear allocation concealment, lack of blinding, or high attrition. ^b^ Downgraded one level due to imprecision: confidence intervals include both important benefit and harm. ^c^ Downgraded two levels due to indirectness and risk of bias: only one small observational study with potential confounding. Note: Hemodynamic outcomes from a single observational study with 9 participants were excluded from the SoF table due to very low certainty and limited applicability. These results are described narratively in the main text.

## Data Availability

The data supporting the results of this study are available at reasonable request to the corresponding author. Due to privacy and ethics restrictions, the data are not publicly available. However, aggregated summary data are included in the manuscript and Supplementary Materials.

## Supplementary Materials

The following supporting information can be downloaded at https://www.mdpi.com/article/10.3390/medicines12040023/s1, PRISMA 2020 Checklist.

## Abbreviations

The following abbreviations are used in this manuscript:

HFrEF	Heart Failure with Reduced Ejection Fraction
HFpEF	Heart Failure with Preserved Ejection Fraction
LVEF	Left Ventricular Ejection Fraction
PCWP	Pulmonary Capillary Wedge Pressure
CI	Cardiac Index
NT-proBNP	N-terminal pro-B-type Natriuretic Peptide
SVI	Stroke Volume Index
RoB-2	Risk of Bias 2
JBI	Joanna Briggs Institute
LVAD	Left Ventricular Assist Device
COPD	Chronic Obstructive Pulmonary Disease
PRISMA	Preferred Reporting Items for Systematic Reviews and Meta-Analyses
PROSPERO	International Prospective Register of Systematic Reviews
AE	Adverse Events
VT	Ventricular Tachycardia
AF	Atrial Fibrillation
SVT	Supraventricular Tachycardia
RRT	Renal Replacement Therapy
CABG	Coronary Artery Bypass Grafting
HF	Heart failure
cTnC	Cardiac troponin C
cAMP	Cyclic Adenosine Monophosphate
ATP	Adenosine Triphosphate
eGFR	Estimated Glomerular Filtration Rate

## Appendix A

**Table A1:** Selected databases, keywords, search strategies, and filters used in the initial article retrieval process.

Date Base	Keywords	Search Strategy	Filters Applied in the Database	Number of Possible Items to Select
PubMed/Medline	Levosimendan, Simdax, Heart Failure with Reduced Ejection Fraction, HFrEF, Older Adults, Elderly, Hemodynamics, Cardiac Output, Left Ventricular Function	(Levosimendan) AND (Heart Failure, Systolic [MeSH] OR Heart Failure with Reduced Ejection Fraction OR HFrEF) AND (Older Adults OR Elderly) AND (Randomized Controlled Trial [Publication Type] OR Clinical Trial OR Observational Study) AND (Hemodynamics [MeSH] OR Cardiac Output OR Left Ventricular Function)	Systematic Reviews and Meta-Analyses, Clinical Trials, and Observational Studies, published in the Last 20 Years.	16
ScienceDirect	Levosimendan AND Heart Failure with Reduced Ejection Fraction AND Older Adults	“Levosimendan AND Heart Failure with Reduced Ejection Fraction AND Older Adults”	Research Articles and Review Articles published in the Last 20 Years.	349
Scopus	Levosimendan, HFrEF, Elderly, Hemodynamics, Clinical Trials, Observational Studies	“Levosimendan” AND “Heart Failure with Reduced Ejection Fraction” AND (“Older Adults” OR “Elderly”)	Research Articles and Review Articles published in the Last 20 Years.	5
Cochrane Library	Levosimendan, Heart Failure with Reduced Ejection Fraction, Elderly, Systematic Reviews, Meta-Analysis	“Levosimendan” AND “Heart Failure with Reduced Ejection Fraction”	Clinical Trials, Observational Studies, Systematic Reviews, and Meta-analyses published in the Last 20 Years.	9
